# Influence of Pulsed Electric Field and Ohmic Heating Pretreatments on Enzyme and Antioxidant Activity of Fruit and Vegetable Juices

**DOI:** 10.3390/foods8070247

**Published:** 2019-07-08

**Authors:** Cinzia Mannozzi, Kamon Rompoonpol, Thomas Fauster, Urszula Tylewicz, Santina Romani, Marco Dalla Rosa, Henry Jaeger

**Affiliations:** 1 Institute of Food Technology, University of Natural Resources and Life Sciences (BOKU), Muthgasse 18, 1190 Vienna, Austria; 2 Department of Agricultural and Food Science, University of Bologna, Piazza Goidanich 60, 47521 Cesena, Italy

**Keywords:** PEF, OH, POD, colour, extraction

## Abstract

The objective of this work was to optimize pulsed electric field (PEF) or ohmic heating (OH) application for carrot and apple mashes treatment at different preheating temperatures (40, 60 or 80 °C). The effect of tissue disintegration on the properties of recovered juices was quantified, taking into account the colour change, the antioxidant activity and the enzyme activity of peroxidase (POD) in both carrot and apple juice and polyphenol oxidase (PPO) in apple juice. Lower ΔE and an increase of the antioxidant activity were obtained for juice samples treated with temperature at 80 °C with or without PEF and OH pretreatment compared with those of untreated samples. The inactivation by 90% for POD and PPO was achieved when a temperature of 80 °C was applied for both carrot and apple mash. A better retention of plant secondary metabolites from carrot and apple mashes could be achieved by additional PEF or OH application. Obtained results are the basis for the development of targeted processing concepts considering the release, inactivation and retention of ingredients.

## 1. Introduction

Low-intensity pulsed electric field (PEF) can enhance the mass transfer during extraction by increasing cell membrane permeability, known as electroporation. Therefore, PEF treatments can enhance the release of specific intracellular compounds from plant tissues [1,2,3,4].

At the same time, ohmic heating (OH) as an alternative thermal pretreatment could be used prior to extraction. The volumetric energy dissipation and the rapid and uniform heating represent advantages, especially for viscous particulate products such as fruit or vegetable mash [5]. In addition, the short processing times during the OH treatment may cause less degradation of colour- and heat-sensitive substances.

In plant cells, antioxidant compounds are mainly located in the vacuoles, whereas the enzymes peroxidase (POD) and polyphenoloxidase (PPO) are found in plastids [6].

Processing of plant tissues compromises the internal compartmentalization that allows the contact between degradative enzymes and their substrates (phenolic compounds), implying the reaction known as enzymatic browning. In the case of POD, phenolic compounds are oxidized at the expense of H_2_O_2_, leading to flavour changes [7]. Instead, PPO is an oxidoreductase which catalyses the oxidation of phenolic compounds in o-quinones, which are subsequently polymerized into brown pigments [8]. Therefore, the inactivation of POD and PPO enzymes is a crucial prerequisite and indicator of quality in the processing of fruit and vegetables.

Moreover, the activation of enzymes, including an increased release and the enhancement of enzymatic reactions by the cell disintegration applied at early stages during fruit and vegetable processing might be a limitation for the shelf-life of recovered juices. Thermal treatment has been used in order to reduce the enzyme activity, but it causes negative effects on quality and related nutritional compounds [9]. Nonthermal food preservation technologies are considered to be more efficient in terms of required energy and in terms of avoiding heat-induced changes of colour, flavour and nutritional value [10]. However, enzyme inactivation achieved during the nonthermal preservation of juices is rather limited [11].

Carrot and apple are good sources of carotenoids and phenols, which are located in the chromoplasts and in the vacuoles of the plant cells, respectively [12,13]. They are considered to provide health benefits due to the antioxidant properties that also contribute to the colour and sensory quality of fresh and processed products [14].

To promote the selectivity of the extraction of bioactive compounds from plant tissues, pulsed electric field [15,16,17] and ohmic heating [18,19] treatment have been already investigated.

Bhat et al. [18] applied thermal and OH treatment (60–90 °C; 1–5 min) to bottle gourd and compared the effects on total phenolic content and colour of obtained juices. The total phenolic content increased with OH and thermal application at 80 °C for 4 min and 90 °C for 5 min, respectively, and the best colour retention was observed for OH-treated juice at 80 and 90 °C.

Saxena et al. [20] reported the effect of OH treatment on PPO activity in sugarcane juice. A high PPO inactivation was observed by applying 32 V/cm at 90 °C for 5 min.

However, for the optimization of the PEF and OH process parameters with regard to reducing the energy requirement and process time and increasing yield and quality, more information is required. Subsequently, the impact on enzyme activity and the recovery of bioactive compounds from the raw material need to be investigated. A first part of the study focused on juice yield and selected ingredients extraction by taking into account the induced cell disintegration effects [21]. This second part of the work aimed at understanding the effects of PEF and OH treatments on antioxidant properties, colour and enzyme activity such as peroxidase (POD) for both juices and polyphenoloxidase (PPO) for apple juice. The optimization of the two processing technologies was performed by the modulation of process parameters as well as treatment temperatures, by applying a preheating step (40, 60 or 80 °C) in order to evaluate the effects of thermal and electric field on antioxidant and enzyme activity of recovered apple and carrot juices.

## 2. Materials and Methods

### 2.1. Plant Raw Material and Mash Preparation 

Raw materials (carrots and apples) were bought from the local market in Vienna (Austria). They were washed and cut into smaller pieces. A mill (Alexanderwerk, Remscheid, Germany) with replaceable stainless-steel screens with a grinding level of 2 mm for carrots and 5 mm for apples was used in order to produce the mash.

### 2.2. Mash Pretreatment and Juice Production

For PEF treatment of apple and carrot mash, a batch PEF system (DIL, Quakenbrück, Germany) was used. The distance between parallel plate electrodes in the treatment chamber was set to 5 cm. 50 exponential decay pulses (discharge capacity 0.5 µF, pulse energy 4 J, pulse width 10 µs) were applied to 400 g of mash. The output voltage was set to 4 kV in order to achieve in the treatment chamber an electric field strength of 0.8 kV/cm. A total specific energy input (Wspecific) of 0.5 kJ/kg was applied. The total treatment time of 0.5 ms was calculated by multiplying the pulse width with the number of pulses. 

OH treatment was performed in the same treatment chamber as for the PEF treatment by using a generator (DIL, Germany) to apply the electric field (1.1 A, 572 V, 12 kHz, 0.6 kW), resulting in an electric field of 114 V/cm. Different temperature–time profiles were acquired depending on the selected temperatures for the different carrot and apple mashes [21].

For preheating to the different initial temperatures (40, 60 and 80 °C), microwave (MT 267, Whirlpool, München, Germany) heating with a power of 850 W was applied for different predefined times. Temperatures were measured with a PT100 thermocouple directly during the treatment [21].

After the different pretreatment, all mash batches were cooled to room temperature and pressed using a manual laboratory juice press (Hafico, Germany) with textile cloth; eleven juice samples were obtained for both carrot and apple, in three replicates each (Table 1).

The obtained juices were evaluated regarding different analytical parameters. Colour measurement was performed directly in the fresh juice. For the determination of antioxidant activity (DPPH and ABTS method) and enzymatic activity such as peroxidase (POD) for both juices and polyphenol oxidase (PPO) for apple, juice samples were preserved in the frozen storage (−30 °C) until their use for the analysis. 

### 2.3. Colour Measurements

Juice colour was measured using a Digieye colour measurement system (Verivide, UK). For each juice sample, L*, a* and b* parameters from CIELAB scale were measured. Total colour difference ΔE between untreated and treated juice samples and browning index (BI, for apple juice only) were calculated by equations (1) and (2), respectively. It has to be stated that the untreated juice showed a high degree of colour change due to oxidation and enzymatic browning. Hence, larger ΔE values, i.e., larger deviations from the untreated juice represent the preferred colour for high-quality juices.

(1) $$\Delta E=\sqrt{\Delta L^{2}+\Delta a^{2}+\Delta b^{2}},$$

(2) $$BI=\frac{\left[(100\left(x-0.31\right)\right]}{0.172}$$

where:

(3) x=(a+1.75L)/(5.645L+a−3.012b)

The colour analyses were carried out in fifteen repetitions from each carrot and apple juice sample. 

### 2.4. Antioxidant Activity (DPPH and ABTS Method)

The carrot and apple juices were centrifuged for 15 min at 10000 × g in a centrifuge (Eppendorf, Germany). The supernatants were for the antioxidant activity analysis by two methods, DPPH and ABTS.

The DPPH scavenging activity was evaluated according to [22]. A spectrophotometer (Photometer model U-1100, Hitachi, Ltd. Tokyo, Japan) set at wavelength of 517 nm was used to measure the absorbance. Quantification of the antioxidant activity was made by plotting a Trolox calibration curve (r^2^ = 0.9880), and the results were expressed as mmol Trolox/L of juice.

The ABTS+▪ scavenging activity was carried out as described by [23]. The absorbance was measured with a spectrophotometer (Photometer model U-1100, Hitachi, Ltd. Tokyo, Japan) at 734 nm for a total time of 6 min. The results were expressed as mmol Trolox/L of juice by the quantification with Trolox standard curve (r^2^ = 0.9946). 

The values obtained are the average of three replicates from each juice sample.

### 2.5. Enzyme Activity

#### 2.5.1. POD Assay

Carrot and apple juices were centrifuged at 10,000 × g and 4 °C for 15 min. The supernatant was collected and analysed for POD activity at 470 nm and 25 °C, as described by [24]. The enzymatic extract was obtained by mixing 6.25 mL of juice sample with 12.5 mL of cold potassium phosphate buffer 0.1 M (pH 6.5) for 2 min. The POD substrate solution was prepared by mixing 0.1 mL of 99.5% of guaiacol, 0.1 mL of 30% of hydrogen peroxide and 99.8 mL of 0.1 M of potassium phosphate buffer (pH 6.5). POD activity was assessed by adding 150 µL of enzymatic extract to 3.35 mL of substrate solution in 10 mm pathlength glass cuvettes. POD activity for carrot and apple juice was calculated based on the slope (ΔA/min) of the linear portion of the plot of absorbance compared with time. An enzyme unit is defined as the enzyme activity that catalyses the conversion of 1 µmol of substrate into product in one minute.

#### 2.5.2. PPO Assay

4-Methylcatechol 80 mM prepared in Mcllvaine’s buffer solution at pH 7.5 was used as substrate, and 12.5 mL of cold Mcllvaine’s buffer solution at pH 7.5 was added to 6.25 mL of enzymatic extract. PPO activity for apple juice was determined by reading the absorbance at 420 nm and 25 °C and calculated on the basis of the slope of the linear portion of the curve (ΔA/min). An enzyme unit is defined as the enzyme activity that catalyses the conversion of 1 µmol of substrate into product in one minute.

### 2.6. Data Analyses

The obtained data were analysed using parametric analysis of variance (ANOVA) using Tukey’s HSD post-hoc test, performed with confidence level (*p* < 0.05). Conversely, when the normality of the distribution and the homogeneity of the variances were not satisfied, nonparametric ANOVA (Kruskal–Wallis) along with Holm’s post-hoc tests were carried out (*p* < 0.05). All the statistical analyses were performed by using R statistical software R x64 3.4.3 (R foundation for statistical computing, Vienna, Austria).

## 3. Results and Discussion

### 3.1. Colour

**Figure 1:**
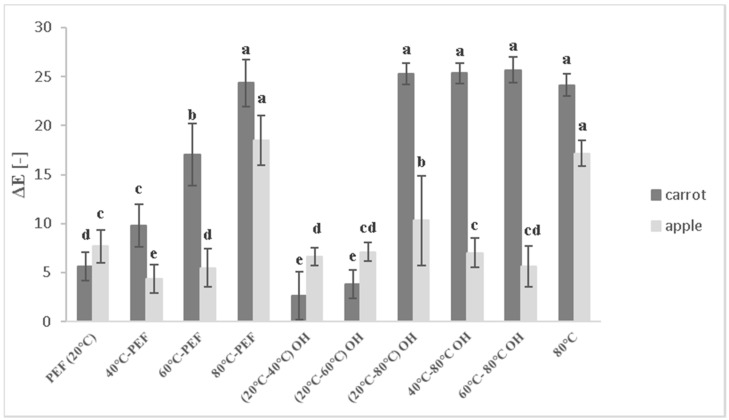
Total colour variation—ΔE between apple and carrot juices obtained from untreated and treated mash. Different letters indicate significant differences (*p* < 0.05) between samples.

Colour changes represent an indicator for enzymatic browning, as well as for process-induced browning due to heat-induced formation of Maillard products. Total colour variation (ΔE) between untreated and treated carrot and apple juice samples was analysed and is shown in Figure 1.

Larger ΔE values represent a positive deviation from the untreated control sample that showed undesired browning due to enzyme activity and oxidation. 

Juices from both raw materials pretreated at 80 °C with or without PEF showed higher ΔE values compared with those of control samples. According to the classification of [25], ΔE changes above 6 indicate great visible changes. The increase in ΔE reflects the increase in the lightness and decrease in the a* value of samples [26]. Since the untreated juice, which is considered as control sample in this case, showed unwanted browning and colour change due to enzyme activity and oxidation, higher ΔE values, i.e., higher deviation from the control juice colour indicated beneficial quality.

Lower ΔE values were observed for mash pretreated with PEF at room temperature and at 40 and 60 °C with OH.

The lowest ΔE values (4.36–5.49) were observed for preheated juice samples at 40 °C and 60 °C coupled with PEF, and the highest total colour differences were observed for samples preheated to 80 °C with or without additional PEF treatment (18.49 and 17.15, respectively).

**Figure 2:**
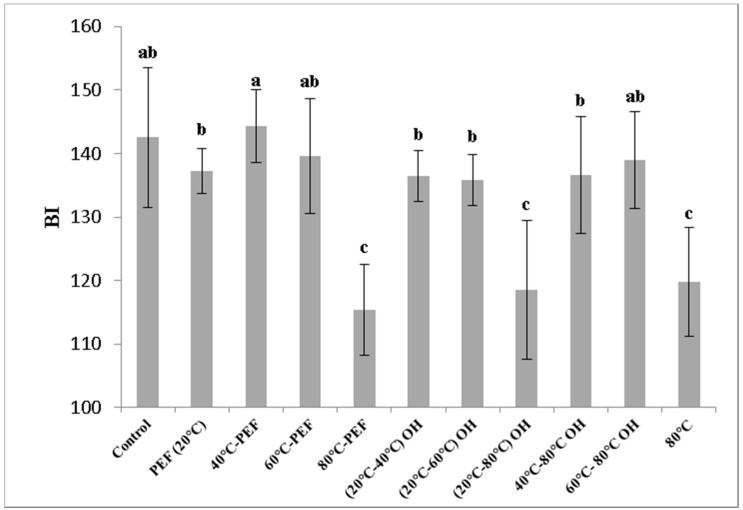
Browning index—BI in apple juice obtained from pretreated apple mash. Different letters indicate significant differences (*p* < 0.05) between samples. pulsed electric field (PEF); ohmic heating (OH).

The detected ΔE values between untreated and treated samples were even more pronounced for carrot compared with those for apple juice. In general, for both juices, higher L* values promoted also higher total colour differences compared with those of the control one. The browning index (BI) is a common parameter to describe colour and juice quality for apple. A decrease of the BI was found for apple samples preheated at 80 °C, coupled with or without PEF or OH treatment, in which was observed to reach BI from 115 to 119, compared with the untreated juice with much higher values of 142 (Figure 2).

Bhat et al. [18] reported similar results for bottle gourd treated with OH at 80 °C for 1 and 2 min (BI of 111 and 101, respectively). The progressive decrease in BI with increasing treatment temperature in apple mash indicates the relevance of enzymatic browning in untreated samples and the role of the temperature during mash treatment for the avoidance of unwanted reactions.

The main groups of pigments that are responsible for the characteristic colours in fruits and vegetables are carotenes and carotenoids, anthocyanins, chlorophylls and phenolic compounds. The main enzymes involved in biochemical degradations of plant compounds are peroxidase and polyphenoloxidase [6]. Moreover, another main cause of brown colour formation is nonenzymatic browning occurring in vegetable and fruit products. However, in the current study, the benefit from short-time thermal treatment of the juice of up to 80 °C for the inactivation of oxidative enzymes was more pronounced than the occurrence of detrimental colour changes due to nonenzymatic browning.

### 3.2. Antioxidant Activity (DPPH and ABTS Method)

**Figure 3:**
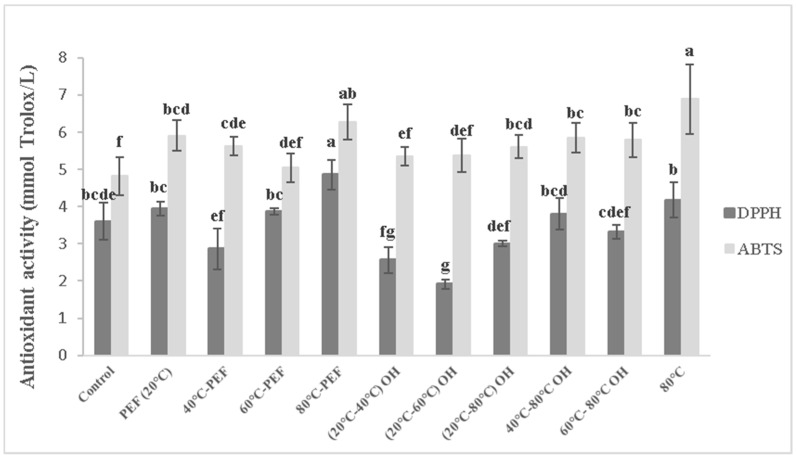
Antioxidant activity (DPPH and ABTS method) of carrot juices obtained from pretreated mash. Different letters indicate significant differences (*p* < 0.05) between samples.

**Figure 4:**
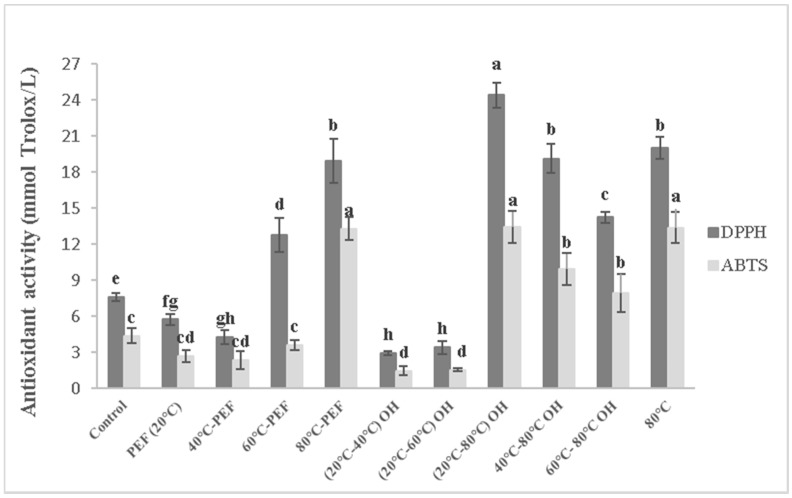
Antioxidant activity (DPPH and ABTS method) of apple juices obtained from pretreated mash. Different letters indicate significant differences (*p* < 0.05) between samples.

Figure 3 and Figure 4 report the results of antioxidant activity, obtained with DPPH and ABTS antiradical activity methods, of differently obtained carrot and apple juices, respectively.

For carrot juice (Figure 3), a significantly higher antioxidant activity was obtained for carrot juices preheated to 80 °C with or without additional PEF or OH treatment with the ABTS method. Higher retention of bioactive compounds with DPPH method was observed for carrot mash pretreated at 80 °C coupled with PEF treatment. However, detected with DPPH method, the application of OH treatment reaching 40 and 60 °C reduced the antioxidant activity, in comparison with that of juice from the untreated control carrot mash. With ABTS method, no significant difference was found (Figure 3).

Significantly higher antioxidant activity, detected with both DPPH and ABTS methods, was obtained for apple juices preheated to 80 °C with or without additional PEF or OH treatment (Figure 4). Instead, OH reaching 40 °C and 60 °C reduced the antioxidant activity, with both used method, compared with that of the apple control juices, which might be due to the activation of degradative enzymes, such as peroxidase and polyphenoloxidase that lead to bioactive compounds’ oxidative degradation.

Fruits and vegetables are good sources of natural antioxidants, containing carotenoids, vitamins, phenolic compounds, flavonoids, dietary glutathione and endogenous metabolites. However, the majority of the antioxidant activity of fruits and vegetables is derived from phenolic compounds (hydroxycinnamic acids, flavan-3-ols, anthocyanidins, flavonols and dihydrochalcones) rather than vitamin C and E, or β-carotene, due to their stronger activity against peroxil [27]. The peroxidase and polyphenoloxidase enzymes lead to the degradation of phenolic compounds and a subsequent loss of nutritional and sensorial values such as browning and off-flavour [28]. Moreover, heat treatment may influence the binding properties of bioactive compounds causing their higher release, but at the same time increase enzymatic or nonenzymatic degradation processes that can cause subsequent negative effects on quality of processed products [15].

In fact, higher temperature leads to the inactivation of the oxidative enzymes, thus reducing degradation effects and resulting in higher antioxidant activity in the juice. Moreover, additional effects other than those from thermal treatment need to be taken into account, since the electropermeabilization, induced by PEF and OH treatment, may contribute to the increased release of antioxidant compounds.

The detected difference between the two different methods used could be due to the fact that DPPH method is more sensitive to detect flavanones, while ABTS for the radical scavengers such as vitamin C [29]. 

For carrot and apple juice, PEF treatment without preheating did also not affect the extractability of bioactive compounds, which is in accordance with Shilling et al. [15], who reported no significant differences on total antioxidant activity between control and PEF-treated apple mash for different electric fields (1, 3 and 5 kV/cm).

### 3.3. Enzyme Activity

#### 3.3.1. POD Activity

Process pretreatment for the juice production is an important operation in order to improve the quality of the vegetable and fruit raw materials as well as to avoid the activation of degradative enzymes such as POD and PPO that consequently provoke pigments and nutrients loss [30].

**Figure 5:**
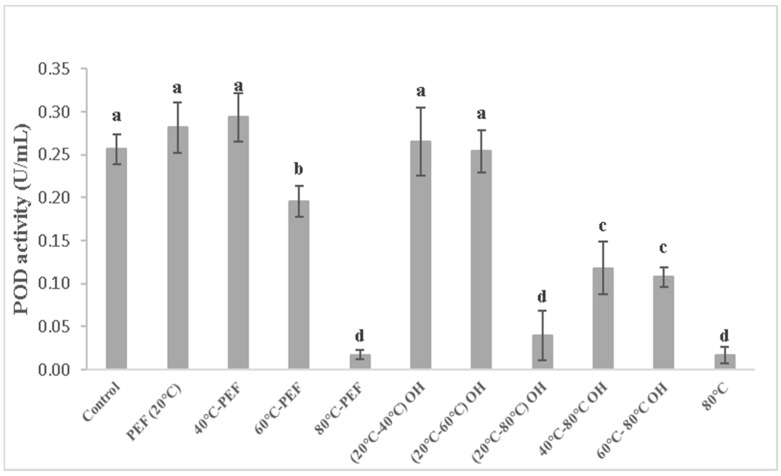
Peroxidase (POD) activity in carrot juice obtained from pretreated mash. Different letters indicate significant differences (*p* < 0.05) between samples.

Peroxidase (POD) activity for differently obtained carrot juice is shown in Figure 5.

This study revealed that the effect of only PEF treatment at 20 °C and 40 °C preheating could not reduce the activity of POD in both carrot (Figure 5) and apple juice [26], while for the samples preheated at 60°C, the reduction of POD activity was observed.

The highest POD inactivation could be reached by preheating to 80 °C with and without additional PEF or OH treatment. All pretreatment conditions with temperature at or up to 60 °C and 40–80 °C by OH treatment led to a decrease in POD activity in the carrot juice, while for apple juices, a greater reduction of the activity (from 50% to 90%) was achieved by 60 and 80 °C preheating temperatures with and without additional PEF or OH application. Enzyme inactivation in the juice, after higher PEF treatment intensities, for microbial inactivation and preservation purposes, is mainly related to secondary effects such as local temperature distributions, electrochemical reactions or formation of free radicals, instead of primary effects of electric field. For the treatment of mash, the PEF treatment intensity can be considered 10-fold lower and having no direct effect on fruit and vegetable mash ingredients. 

POD activity decreased with increasing temperature, and almost no POD activity was detected in juice extracted when the treatment temperature reached 80 °C, especially in apple juice samples. High temperature leads to an increase in the internal energy of the enzymes, thus consequently causes the break of bonds that determine the three-dimensional structure of enzymes [18].

Moreover, with the increasing of temperature, the enzyme activity decreased and required a particular temperature–time combination for complete inactivation. Inadequate temperature led to a decrease in the enzyme activity time rather than complete inactivation, which may cause browning effect. In fact, Bhat et al. [18] reported similar results for OH-treated bottle gourd juice, where the temperature of 60 and 70 °C seemed to be not enough for complete enzyme inactivation, which instead was observed at 80 °C for 4 min.

Icier et al. [31] showed that OH treatment could be used for POD inactivation on pea puree at the range of 30–50 V/cm combined with the water blanching. Elez-Martínez et al. [32] reported a completely POD deactivation in orange juice after the application of PEF treatment at 35 kV/cm for 1500 µs. 

Moreover, the variations of colour for carrot and apple juice pretreated with 80 °C with both PEF and OH applications could be explained by the decrease of enzyme activity, in fact, a correlation between colour and POD activity was found to be 0.8902 and 0.5166, respectively, for carrot and apple juice.

#### 3.3.2. PPO Activity

**Figure 6:**
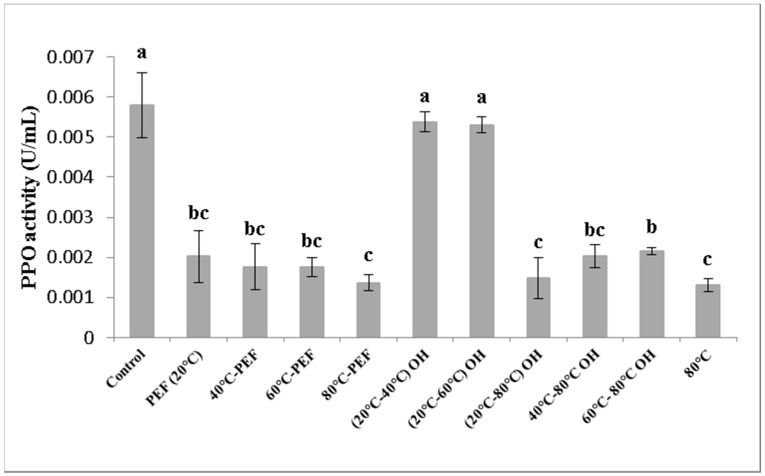
Polyphenoloxidase (PPO) activity of apple juice obtained from pretreated mash. Different letters indicate significant differences (*p* < 0.05) between samples.

Polyphenoloxidase (PPO) activity of differently obtained apple juice samples is shown in Figure 6.

PPO is an oxidoreductase enzyme which catalyses the oxidation of phenolic compounds in o-quinones, which are subsequently polymerized into brown pigments [33]. Heating treatment seems to be the most effective applied treatment for the stabilization of food products against microbial and enzyme activity. Nevertheless, thermal treatment has been shown to cause negative effects on quality and related nutritional compounds [9]. The mechanism of enzyme inactivation is not completely clear. Current results show empirical proof of protein modification by electrical fields [34] that may provoke a deformation or structural change of a protein, due to the interaction between the external electric field and the functional groups of the protein that allow its unfolding [32]. 

PPO activity was significantly decreased by PEF application at room temperature compared with that of the untreated control sample. In addition, a greater inactivation was achieved when the treatment temperature reached 80 °C, as well as with OH treatment and just preheating. Moreover, PPO inactivation was even more effective when a combination of temperatures and PEF or OH applications were used.

Similar results were reported by Turk et al. [35]. PPO activity was reduced in apple cider mash pretreated with PEF at 1 kV/cm for 100 µs. The loss of PPO activity could be explained also by the inhibition of the enzyme with oxidised phenolic compounds, in particular procyanidins [36].

Previous work reported similar results for PPO deactivation; the residual PPO activity was 35% after 14 min with OH treatment at 70 °C, by applying 35 V/cm in grape juice [30].

Liang et al. [37] found a significant decrease (33%) in PPO activity in freshly squeezed apple juice when preheated at 50 °C and treated with PEF at 27 kV/cm for 58.7 µs.

Saxena et al. [20] found a reduction of PPO activity up to 97.8 % by applying 32 V/cm with OH treatment at 90 °C for 5 min in sugarcane juice. Moreover, a greater increase in residual PPO activity was visible at 90 °C by increasing the holding times of OH treatment (5, 10, 15 and 20 min). The increase of the enzyme activity with the holding time at constant temperature was attributed to the pulsating OH treatment that may cause biochemical reactions by changing the molecular spacing and may result in a better interaction between substrate and enzyme [38]. A recent review on the impact of electric fields on enzymes is provided by [39].

## 4. Conclusions

Obtained results emphasize the role of thermal treatment for the inactivation of enzymes, reflected by improved colour values for juices exposed to 80 °C, independent of the PEF or OH application. The inactivation of POD and PPO was more pronounced when a temperature of 80 °C was achieved for both carrot and apple mash (around 90%). 

However, a better retention of plant secondary metabolites from carrot and apple mashes could be achieved by additional PEF or OH application. PEF treatment was found to improve the release of such compounds, whereas OH contributed to a very fast volumetric heating that reduced the overall thermal load that the sample was exposed to. Based on the results, a combination of thermal and electric field pretreatments is required for the controlled release, inactivation and retention of ingredients. Thermal effects contributing to the colour, bioactive compounds retention and enzyme inactivation were found to be still important when applying nonthermal cell disintegration techniques such as PEF. However, both electrotechnologies, PEF and OH were found to positively contribute to improved juice quality by enhanced ingredient release and retention.

## Figures and Tables

**Table 1:** Overview on mash-treatment conditions applied for apple and carrot mash. Pulsed electric field (PEF), ohmic heating (OH).

Treatment	Sample	Wspecific (kJ/kg)
Untreated	Control	0
PEF at 20 °C	PEF (20 °C)	0.5
Preheating 40 °C + PEF	40 °C-PEF	192.5
Preheating 60 °C + PEF	60 °C-PEF	382.5
Preheating 80 °C + PEF	80 °C-PEF	765.5
OH from 20 °C to 40 °C	(20 °C–40 °C) OH	110
OH from 20 °C to 60 °C	(20 °C–60 °C) OH	222
OH from 20 °C to 80 °C	(20 °C–80 °C) OH	355
Preheating 40 °C + OH to 80 °C	40 °C–80 °C OH	402.5
Preheating 60 °C + OH to 80 °C	60 °C–80 °C OH	497.5
Preheating 80 °C	80 °C	765
